# Estrogen Receptor Alpha and Nuclear Factor Y Coordinately Regulate the Transcription of the SUMO-Conjugating *UBC9* Gene in MCF-7 Breast Cancer Cells

**DOI:** 10.1371/journal.pone.0075695

**Published:** 2013-09-27

**Authors:** Shibo Ying, Thomas Dünnebier, Jing Si, Ute Hamann

**Affiliations:** Molecular Genetics of Breast Cancer, Deutsches Krebsforschungszentrum (DKFZ), Heidelberg, Germany; University of Saarland Medical School, Germany

## Abstract

*UBC9* encodes a protein that conjugates small ubiquitin-related modifier (SUMO) to target proteins thereby changing their functions. Recently, it was noted that UBC9 expression and activity play a role in breast tumorigenesis and response to anticancer drugs. However, the underlying mechanism is poorly understood. To investigate the transcriptional regulation of the *UBC9* gene, we identified and characterized its promoter and *cis*-elements. Promoter activity was tested using luciferase reporter assays. The binding of transcription factors to the promoter was detected by chromatin immunoprecipitation (ChIP), and their functional role was confirmed by siRNA knockdown. *UBC9* mRNA and protein levels were measured by quantitative reverse transcription PCR and Western blot analysis, respectively. An increased expression of *UBC9* mRNA and protein was found in MCF-7 breast cancer cells treated with 17β-estradiol (E_2_). Analysis of various deletion mutants revealed a 137 bp fragment upstream of the transcription initiation site to be sufficient for reporter gene transcription. Mutations of putative estrogen receptor α (ER-α) (one imperfect estrogen response element, ERE) and/or nuclear factor Y (NF-Y) binding sites (two CCAAT boxes) markedly reduced promoter activity. Similar results were obtained in ER-negative MDA-MB-231 cells except that the ERE mutation did not affect promoter activity. Additionally, promoter activity was stimulated upon E_2_ treatment and overexpression of ER-α or NF-YA in MCF-7 cells. ChIP confirmed direct binding of both transcription factors to the *UBC9* promoter *in vivo*. Furthermore, *UBC9* expression was diminished by ER-α and NF-Y siRNAs on the mRNA and protein levels. In conclusion, we identified the proximal *UBC9* promoter and provided evidence that ER-α and NF-Y regulate *UBC9* expression on the transcriptional level in response to E_2_ in MCF-7 cells. These findings may contribute to a better understanding of the regulation of UBC9 in ER-positive breast cancer and be useful for the development of cancer therapies targeting UBC9.

## Introduction

Reversible attachment of small ubiquitin-related modifiers (SUMO) is an important post-translational protein modification in eukaryotic cells [1,2]. Substrate modification by SUMOylation can alter protein–protein interactions, change protein intracellular localization or direct changes in the activities of the protein to which SUMO is attached. Mammals typically express three SUMO variants (SUMO 1-3), which are conjugated to substrates through an enzymatic cascade involving the sequential action of the E1 SAE1/SAE2 activating enzyme, the E2 conjugating enzyme UBC9 and several E3 ligases such as the protein inhibitors of activated STAT (PIAS) family proteins that confer substrate specificity [2]. This regulation is dynamic, because it is a highly reversible process due to several SUMO-specific isopeptidases that remove SUMO from targets [3,4]. It is noteworthy that UBC9 is the only E2 conjugating enzyme and therefore a key regulator of the SUMOylation machinery.

There is increasing evidence that deregulation of UBC9 resulting in alterations in SUMOylation affects cancer development, including breast cancer. First, several cellular regulatory proteins are modified by SUMO including important tumor suppressors and oncoproteins, such as PML, WRN, BLM, c-JUN, c-FOS, TP53, MDM2 and EZH2 [5–13]. SUMO also targets several nuclear hormone receptors, including ER-α, progesterone receptor and androgen receptor [14–16], which play a central role in the development of hormone-driven breast tumors, and coregulators of these receptors, thereby modulating their ability to interact with the nuclear receptor and to activate transcription [17–19]. Second, UBC9 is up-regulated in various human malignancies including lung and ovary cancers and melanoma [20–22]. Also in breast cancers, an approximately 6-fold higher UBC9 expression was observed than in matched normal tissues [23]. UBC9 overexpression increased ER-α-mediated transcriptional activity by SUMOylation, implying a possible synergy between UBC9 and a promoting factor for breast cancer development [16,24]. UBC9 overexpression also increased tumor cell growth and promoted cell invasion and metastasis in a SUMOylation-independent manner [21,25]. Moreover, up-regulation of UBC9 correlated with intrinsic or acquired resistance to anticancer drugs, whereas overexpression of a dominant-negative mutant UBC9 increased the sensitivity of tumor cells to DNA-damaging anticancer drugs such as inhibitors of topoisomerase I and topoisomerase II [26]. Finally, variants in the *UBC9* gene have been shown to be associated with a decreased efficacy of DNA double–strand break repair [27], breast tumor grade [28] and risk of grade 1 breast cancer [29].

Although UBC9 expression was reported to be associated with breast tumorigenesis and drug responsiveness, little is known about the underlying mechanism. In this study we assessed the transcriptional regulation of the *UBC9* gene in MCF-7 and MDA-MB-231 breast cancer cells. We identified the minimal *UBC9* promoter region and provide evidence that it is regulated by ER-α and NF-Y in response to E_2_.

## Materials and Methods

### Cells, chemicals and culture conditions

The human breast cancer MCF-7 (ER-positive) and MDA-MB-231 (ER-negative) cell lines were obtained from the American Type Culture Collection (ATCC, Manassas, VA, USA). Cells were cultured in Dulbecco’s Modified Eagle Medium- high glucose (Invitrogen Corporation, Carlsbad, CA, USA), supplemented with 10% fetal bovine serum or dextran-coated charcoal-treated fetal bovine serum at 37°C in a humidified atmosphere with 5% carbon dioxide. 17β-estradiol (E_2_) was purchased from Sigma-Aldrich (Sigma-Aldrich Corporation, St. Louis, MO, USA), and the anti-estrogen ICI 182,780 (ICI) from Tocris Bioscience, Inc. (Ellisville, MO, USA). MCF-7 cells were treated with 10 nM E_2_ and/or 100 nM ICI dissolved in dimethylsulfoxide.

### *In silico* analysis of the putative UBC9 promoter and its *cis*-acting elements

The 5’-flanking sequence of the human UBC9 gene (Human Genome Nomenclature Committee *UBE2I* ubiquitin-conjugating enzyme E2) on chromosome 16p13.3 was investigated *in silico*. The transcription start site refers to the Ensemble Transcript ID ENST00000325437. The putative proximal promoter and its putative transcription factor binding sites were predicted using the webtool PromoterSweep [30].

### Cloning of the UBC9 promoter and construction of deletion mutants

A *UBC9* promoter-driven luciferase reporter construct was generated by inserting a 2,516 bp fragment containing the 5´-flanking region of the *UBC9* gene from nucleotide positions -2,392 to +124 into the *Kpn*I and *Bgl*II sites of the *pGL*4.10 promoterless vector containing the *firefly* luciferase gene (Promega, Fitchburg, WI, USA). The fragment was amplified from human genomic DNA by PCR using LA PCR kit version 2.1 (Takara, Shiga, Japan) according to the manufacturer’s instructions. The PCR conditions consisted of an initial denaturation at 94°C for 1 minute, followed by 30 cycles of denaturation at 98°C for 10 seconds and annealing at 68°C for 3 minutes. Subsequently, five 5´-deletion mutants, -1,852/+124, -1,310/+124, -404/+124, -137/+124 and -5/+124 were generated by PCR using the full length *UBC9* promoter fragment (-2,392/+124) as a template. The PCR conditions consisted of an initial denaturation at 95°C for 1 minute followed by 30 cycles at 95°C for 30 seconds, annealing at 55°C for 30 seconds and extension at 72°C for 4 minutes, and then one cycle at 72°C for 8 minutes. The five amplified PCR fragments were ligated into the *pGL*4.10.vector. All constructs were sequenced to confirm variant incorporation. Primers used to generate all constructs are listed in Table S1.

### Site-directed mutagenesis

Site-directed mutagenesis using QuikChange site-directed mutagenesis kit (Stratagene, La Jolla, CA, USA) was performed to generate mutations in the putative transcription factor binding sites using pGL-137/+124 as a template. PCR conditions were also according to the manufacturer’s instructions. The mutant constructs were verified by DNA sequencing. Primers used for site-directed mutagenesis are shown in Table S1.

### Transient transfections and luciferase reporter assays

Cells seeded in 96-well plates and grown to a density of 80% (1×10^4^) were transfected with 100 ng of each luciferase reporter construct and 0.5 µl Lipofectamine LTX Reagent (Invitrogen) in serum-free medium according to the manufacturer’s instructions. To correct for transfection efficiency, cells were co-transfected with 4 ng of *pGL*4.74 vector (Promega), which contained the *Renilla* luciferase gene (hRluc) under the control of the Herpes simplex virus thymidine kinase promoter. Co-transfection of cells with NF-YA or ER-α expression vectors was performed using 10 ng of DNA.

Transfected cells were harvested after 48 hours, washed twice with PBS and then lysed with 20 µl of passive lysis buffer per well (Promega). Luciferase activity was measured using a Dual-Luciferase Reporter Assay System (Promega) on a Mithras LB 940 Multimode Microplate Reader (Berthold technologies, Bad Wildbad, Germany). To correct for any differences in transfection efficiency or cell lysate preparation, *Firefly* luciferase activity was normalized to *Renilla* luciferase activity and expressed as ‘

‘fold-induction’’ relative to the empty *pGL*4.10 vector, the activity of which was arbitrarily defined as 1. Four transfections were carried out independently for each construct. Results were expressed as mean ± standard deviation (SD).

### Total RNA extraction and quantitative real-time PCR

Total RNA was extracted and purified from cells using the RNAprotect cell reagent and RNeasy protect cell mini kit (Qiagen, Hilden, Germany) according to the manufacturer’s instructions. Two-step quantitative reverse transcription PCR (RT-PCR) was performed using QuantiTect reverse transcription kit (Qiagen). Subsequently, real-time PCR analysis was carried out using Absolute QPCR SYBR Green Mix (Thermo Scientific, Surrey, UK) according to the manufacturer’s protocol on a LightCycler 480 real-time PCR system (Roche Applied Science, Mannheim, Germany). The amounts of cDNA were normalized to glyceraldehyde-3-phosphate dehydrogenase (GAPDH). Primers used for quantitative real-time PCR are shown in Table S1.

### Chromatin immunoprecipitation

Chromatin immunoprecipitation (ChIP) was performed using the MAGnify chromatin-immunoprecipitation system (Invitrogen) according to the manufacturer’s protocol. Briefly, proteins from cell extracts were cross-linked to DNA by addition of formaldehyde to a final concentration of 1% for 10 minutes at room temperature. Chromatin was sheared by sonication to 200 to 500 bp fragments using the Sonorex RK102H (Bandelin electronic, Berlin, Germany). The soluble chromatin fraction was collected, and 10% of the supernatant was used for input normalization. Equivalent amounts of either anti-NF-YA, anti-ER-α antibodies (Santa Cruz Biotechnology, Santa Cruz, CA, USA) or normal rabbit IgG (negative control, Santa Cruz Biotechnology) were added and incubated according to the protocol. Purified eluted DNA was quantified by quantitative real-time PCR as described above. PCR primer sequences are provided in Table S1.

### siRNA knockdown

For knock-down experiments, MCF-7 cells were co-transfected with the relevant luciferase reporter plasmids and either 100 nM NF-YA-siRNA (human), ER-α-siRNA (human) or Control siRNA-A (Santa Cruz Biotechnology), using the siRNA transfection reagent (Santa Cruz Biotechnology) according to the manufacturer’s instructions. After culturing of cells in antibiotic-free medium in the presence or absence of E_2_ for 48 hours, total RNA was extracted and analyzed by quantitative RT-PCR as described above. To confirm the specific inhibitory activity of each siRNA, Western blot analyses were carried out with antibodies against NF-Y and ER-α as described below.

### Western blot analysis

Total protein from cells was extracted with Qproteome mammalian protein prep kit (Qiagen). Nuclear extracts were prepared from mock or siRNA-transfected cells using the NE-PER Nuclear and Cytoplasmic Extraction Reagents (Thermo Scientific) according to the manufacturer’s instructions. Anti-UBC9 (H-81), anti-Actin (H-196), anti-NF-YA (H-209), anti-ER-α (H-184) or anti-Histone H1 (FL-219) (Santa Cruz Biotechnology), were added in skim milk solution at a dilution of 1:200 each. The secondary anti-rabbit (Santa Cruz Biotechnology) antibody was used at a dilution of 1:3000. Detailed information on the specificity of the antibodies used is given in the data sheets of Santa Cruz Biotechnology Inc. (www.scbt.com). Proteins were detected using Pierce ECL plus Western blotting substrate (Thermo Scientific).

## Results

### UBC9 expression in breast cancer cell lines

In order to evaluate *UBC9* gene expression we investigated *UBC9* mRNA and protein expression in ER-positive MCF-7 and ER-negative MDA-MB-231 breast cancer cells using real-time RT-PCR and Western blot analyses. In MCF-7 cells lower basal *UBC9* mRNA and protein expression levels were observed than in MDA-MB-231 cells (Figure S1).

**Figure 1:**
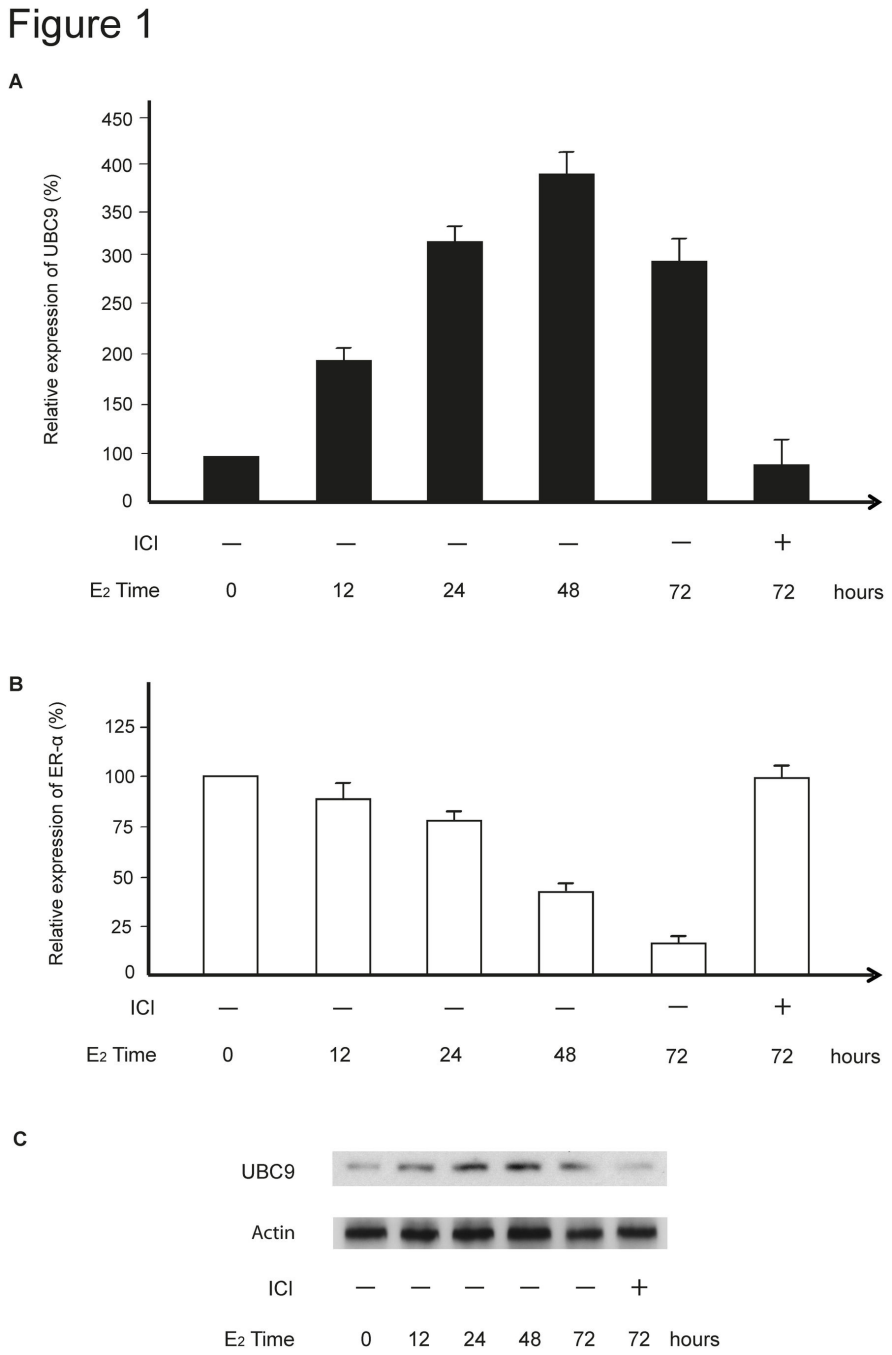
UBC9 expression is up-regulated by E_2_ in MCF-7 cells. (**A**) *UBC9* and (**B**) ER-α mRNA expression after treatment of MCF-7 cells with 10 nM E_2_ for 12, 24, 48 and 72 hours. Where indicated, 100 nM ICI was also added to the culture medium. Total RNA was isolated and analyzed by real-time RT-PCR. Expression levels were normalized to GAPDH expression and relative to expression in untreated cells, which was arbitrarily set to 1. The data refer to results obtained in four separate experiments performed in triplicate. Bars represent the standard deviation (SD). (**C**) Up-regulation of UBC9 protein levels after induction with E_2_. Total protein was extracted and analyzed by Western blotting. Actin was used as an internal protein loading control.

Next we investigated UBC9 and ER-α expression in response to E_2_ in MCF-7 cells. UBC9 mRNA expression levels increased after stimulation with 10 nM E_2_ reaching a maximum after 48 hours (Figure 1A). The expression pattern on the protein level corresponded to that on the mRNA level (Figure 1C). ER-α mRNA expression decreased over time (Figure 1B) and inversely correlated with UBC9 expression levels. Furthermore, the pure anti-estrogen ICI, which is devoid of agonistic activity and produces both *in vivo* and *in vitro* a state of complete estrogen withdrawal [31,32], did not affect ER-α mRNA expression as previously reported [33,34] (Figure 1B), but completely abrogated the E_2_-induced UBC9 expression on both the mRNA and protein levels (Figure 1A and C).

### Identification of the proximal promoter and potential cis-elements

**Figure 2:**
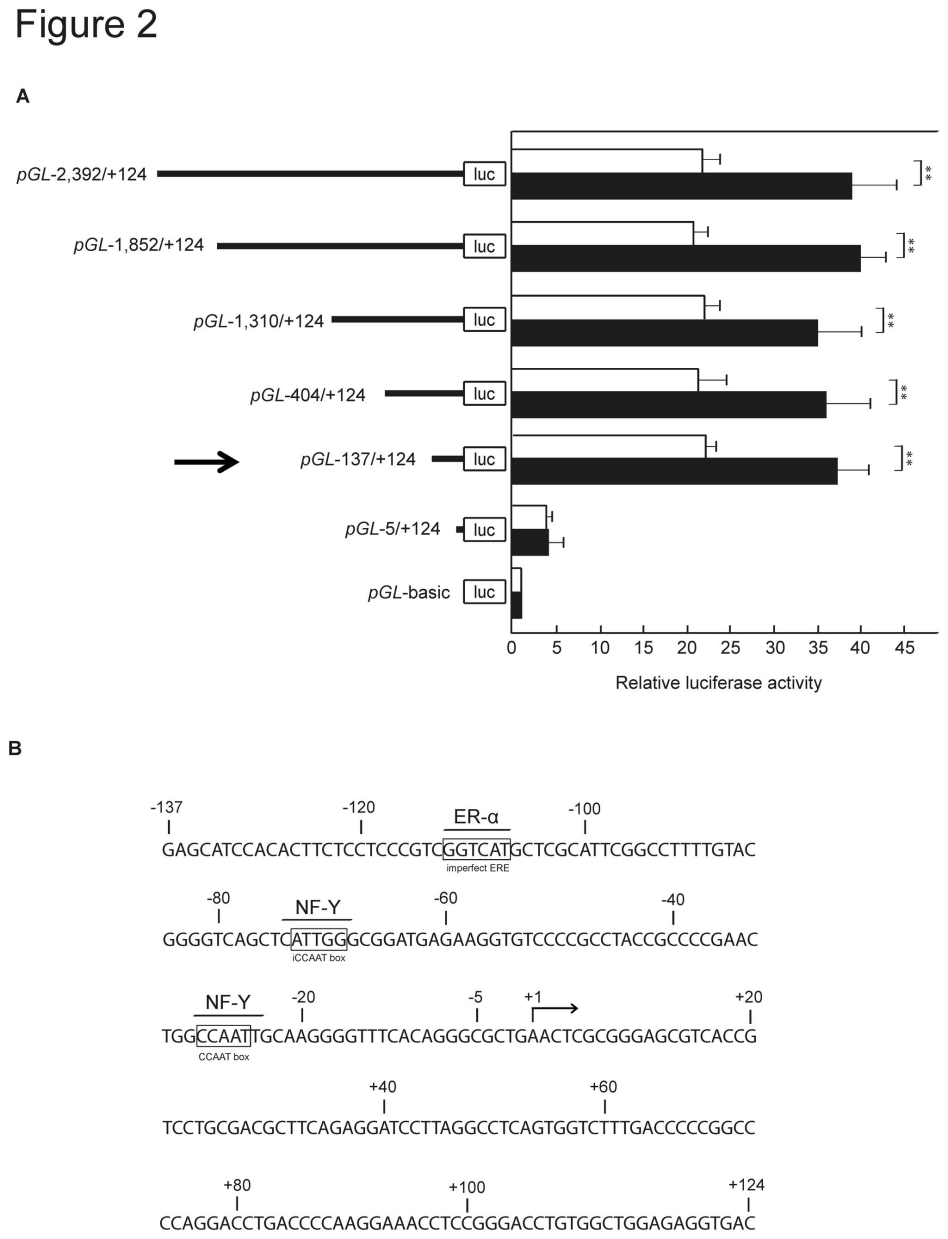
Identification and characterization of the minimal *UBC9* promoter. (**A**) MCF-7 cells cultured in phenol red-free medium in the presence (black bars) or absence (white bars) of E_2_ were transfected with the indicated constructs and assayed for luciferase activity after 48 hours. The numbers given for each construct indicate the 5’ and 3’ ends of the *UBC9* 5’-flanking region; the position numbered +1 corresponds to the transcription initiation site. Luciferase activity was expressed as fold change relative to that obtained from promoter-less vector *pGL*-basic, which was arbitrarily set to 1. Values were normalized for transfection efficiency by co-transfection with the *Renilla* expression plasmid and were given as mean ±SD obtained in four separate experiments. **P<0.01 (Student’s *t*-test). (**B**) *UBC9* sequence and putative transcription factor binding sites of the minimal *UBC9* promoter. Position +1 refers to the transcription initiation site. Putative transcription factor-binding sites predicted by the webtool PromoterSweep [30], including an imperfect ERE, a CCAAT box and an inverted CCAAT box (iCCAAT) are overlined.

To determine the sequence which is sufficient for the transcription of the *UBC9* gene, a 2,516-bp fragment (nucleotides -2,392 to +124) containing the proximal 5’-flanking region and the transcription start site (labelled as +1) was cloned and fused upstream of the promoter-less luciferase reporter gene. In addition, five progressive 5’-deletion mutants of the full-length fragment were generated and examined for their effect on reporter gene activity. Significant transcriptional activities were observed with five constructs: pGL-2,392/+124, pGL-1,852/+124, pGL-1,310/+124, pGL-404/+124 and pGL-137/+124 (Figure 2A). The smallest deletion construct, pGL-5/+124 had decreased transcriptional activity indicating that nucleotides -137 to +124 contain the positive regulatory elements that are essential for basal promoter function.

*In silico* analyses of the *UBC9* 5´-flanking region using the web tool PromoterSweep predicted three *cis*-elements comprising an imperfect ERE GGTCAT at positions -112/-107 for binding of the transcription factor ER-α, an inverted (i) CCAAT box (ATTGG) at positions -73/-69 and a CCAAT box at positions -29/-25, which serve as potential binding sites for nuclear factor Y (NF-Y) (Figure 2B). The presence of multiple putative transcription factor binding sites near the transcription initiation site provides further evidence that nucleotides -137 to +124 may function as the minimal *UBC9* promoter.

### Effect of putative cis-elements on UBC9 promoter activity

**Figure 3:**
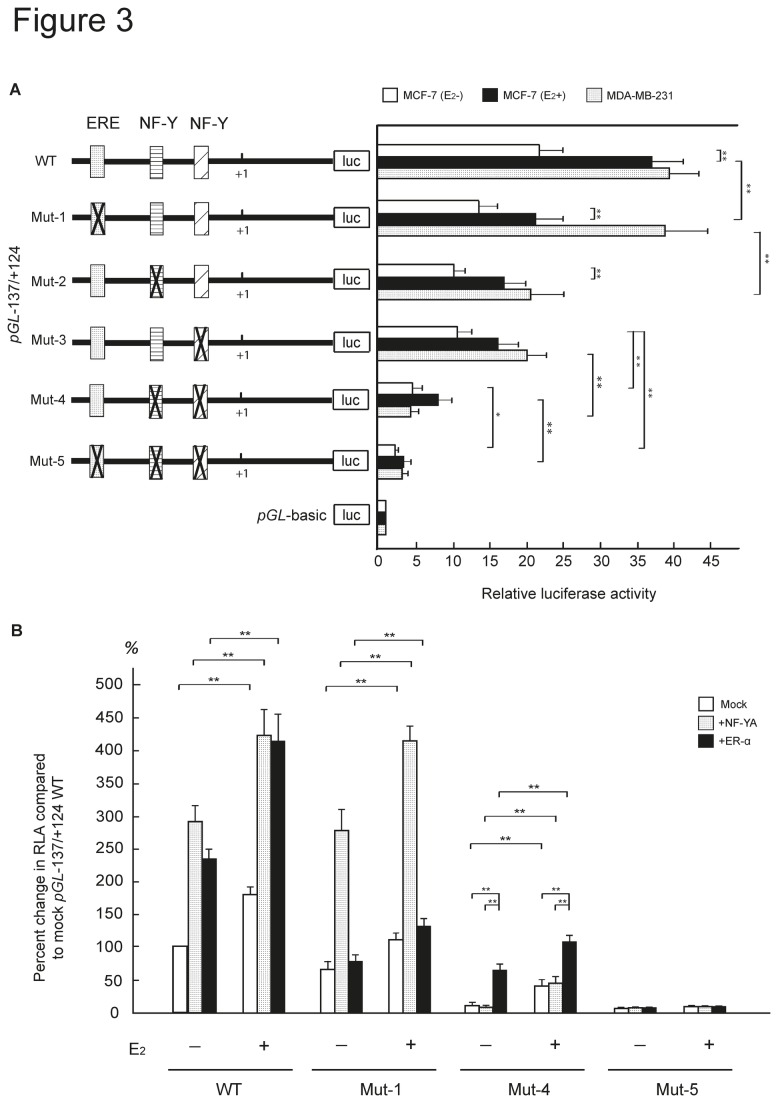
Identification of *cis*-elements of the *UBC* promoter. (**A**) Site-directed mutagenesis was carried out with the UBC9 pGL-137/+124 construct. The mutation of a putative transcription factor binding site is indicated by a solid cross. MCF-7 cells cultured in phenol red-free medium in the absence (white bars) or the presence (black bars) of E_2_ and and MDA-MB-231 cells (dotted bars) were transfected with the indicated constructs and assayed for luciferase activity after 48 hours. Luciferase activity was expressed as fold change relative to that obtained from promoterless vector *pGL*-basic, which was arbitrarily set to 1. Values were normalized for transfection efficiency by co-transfection with the *Renilla* expression plasmid and were expressed as mean ±SD obtained in four separate experiments. *P<0.05, **P<0.01 (Student’s *t*-test). (**B**) Enhancement of the *UBC9* promoter activity by ER-α or NF-YA overexpression. Untreated and E_2_-treated MCF-7 cells were transfected with WT, Mut-1, Mut-4 and Mut-5*pGL*-137/+124 constructs, NF-YA (dotted bars) or ER-α (black bars) expression plasmids. Mock transfected cells were used as a control (white bars). Relative luciferase activity (RLA) was expressed as fold change relative to that obtained from pGL-137/+124 (E_2_-), which was arbitrarily set to 1. Values were normalized for transfection efficiency by co-transfection with the *Renilla* expression plasmid and were expressed as mean ±SD obtained in four separate experiments. **P<0.01 (Student’s *t*-test).

To ascertain the effect of potential *cis*-elements that regulate the transcription of the *UBC9* gene, a series of ER-α and NF-Y transcription factor binding site mutants were generated from the pGL-137/+124 wildtype (WT) construct and transfected into MCF-7 and MDA-MB-231 cells. In MCF-7 cells, mutation of the -112/-107 ERE (Mut-1), the -73/-69 iCCAAT box (Mut-2) and the -29/-25 CCAAT box (Mut-3) resulted in a marked reduction of *UBC9* promoter activity (Figure 3A). An even stronger reduction was observed after mutation of both CCAAT boxes (Mut-4), and promoter activity was nearly abolished after mutation of all three sites (Mut-5) (Figure 3A). E_2_ significantly increased promoter activity of single- and double-site mutants by 30% to 50% compared to untreated MCF-7 cells. This difference was not detected in cells transfected with three-site mutant. In MDA-MB-231 cells similar results were obtained except that Mut-1 had no effect on promoter activity.

To further define the role of ER-α and NF-Y in *UBC9* transcription, the promoter activity was tested in MCF-7 cells transfected with ER-α or NF-Y expression plasmids in the luciferase reporter assay. As expected, overexpression of ER-α or NF-YA

(A: the regulatory subunit of NF-Y) enhanced basal promoter activity of pGL-137/+124WT (Figure 3B). The promoter activity of Mut-4 and Mut-5 constructs was decreased in ER-α or NF-Y overexpressing cells, while that of Mut-1 was only decreased in ER-α overexpressing cells (Figure 3B). Similar to the results of the *cis*-element mutants, E_2_ also strongly stimulated the transcriptional activity of pGL-137/+124WT, Mut-1 and Mut-4 in abundance of ER-α or NF-YA, but not of Mut-5. These results imply that E_2_ not only controls *UBC9* promoter activity via ER-α but also induces NF-YA activity via an E_2_-mediated pathway. Altogether, ER-α- and NF-Y-binding sites within nucleotides -137 to +124 are cooperative *cis*-elements.

### *In vivo* binding of transcription factors to the UBC9 promoter

**Figure 4:**
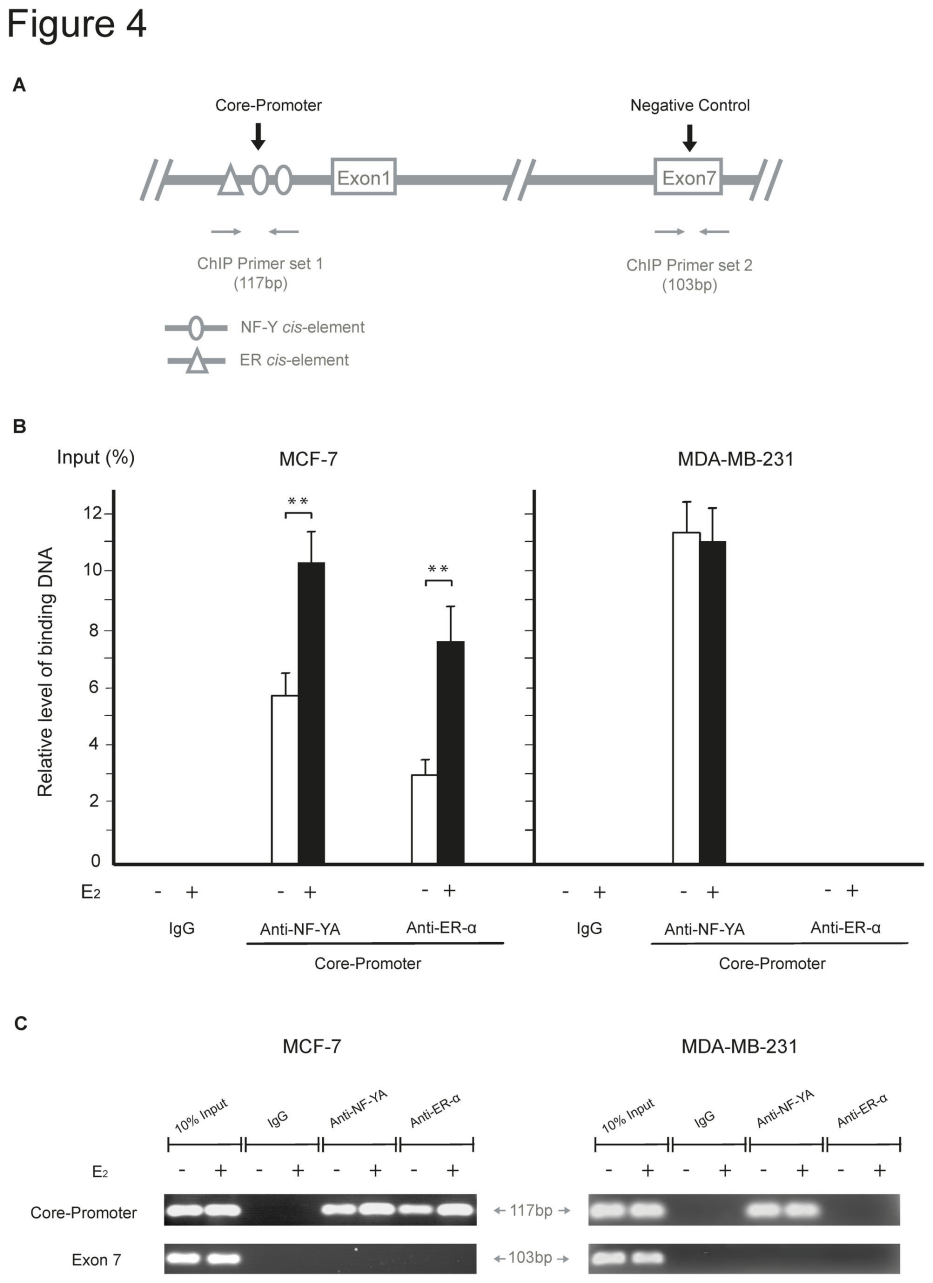
Recruitment of ER-α and NF-Y to the *UBC9* promoter *in vivo*. (**A**) Schematic representation of the *UBC9* gene including the proximal promoter with the putative transcription factor binding sites and the negative control region (*UBC9* exon 7). Primer pairs are indicated by arrows. (**B**) ChIP assays using anti-ER-α, NF-YA or IgG control antibodies were performed on chromatin isolated from cells cultured in phenol red-free medium in the absence (white bars) or the presence (black bars) of E_2_ for 48 hours. The equivalent fraction of the sonicated chromatin was set aside as 'input' DNA (non-immunoprecipitated) before the antibody affinity manipulations. Data were presented as relative amount of immunoprecipitated DNA normalized to input as measured by quantitative PCR assay, and were given as mean ±SD obtained in four separate experiments. **P<0.01 (Student’s *t*-test). (**C**) Ethidium bromide staining of the PCR products of the *UBC9* promoter region (upper panel) and *UBC9* exon 7 control region (lower panel).

To test whether the predicted transcription factors bind to the *UBC9* promoter *in vivo*, we performed chromatin immunoprecipitation (ChIP) using specific anti-ER-α and anti-NF-YA antibodies, specific primers for the *UBC9* promoter region (Table S1) and formaldehyde-fixed chromatin isolated from cultured cells. A schematic representation of the *UBC9* promoter region and its *cis*-acting elements is given in Figure 4A. The binding of the transcription factors was specific in MCF-7 and MDA-MB-231 cells, because no PCR product was detected in chromatin samples immunoprecipitated with non-immune IgG using the same primers (Figure 4B, 4C). The specificity of the ChIP analysis was further demonstrated by the inability to detect binding of ER-α or NF-YA to the *UBC9* exon 7 control region (Figure 4C). In MCF-7 cells, ER-α and NF-YA bound to the 5’-flanking region of *UBC9* (Figure 4B, 4C). In ER-negative MDA-MB-231 cells only an enhanced recruitment of NF-YA to the promoter region was detected (Figure 4B).

After treatment of cells with E_2_ an enhanced ER-α and NF-YA recruitment was observed in MCF-7 cells compared to untreated cells, whereas the level of bound NF-YA remained unchanged in MDA-MB-231 (Figure 4B). These results confirm binding of ER-α and NF-Y to the *UBC9* promoter *in vivo*, which was even increased upon E_2_ treatment.

### Role of ER-α and NF-Y in endogenous *UBC9* expression

**Figure 5:**
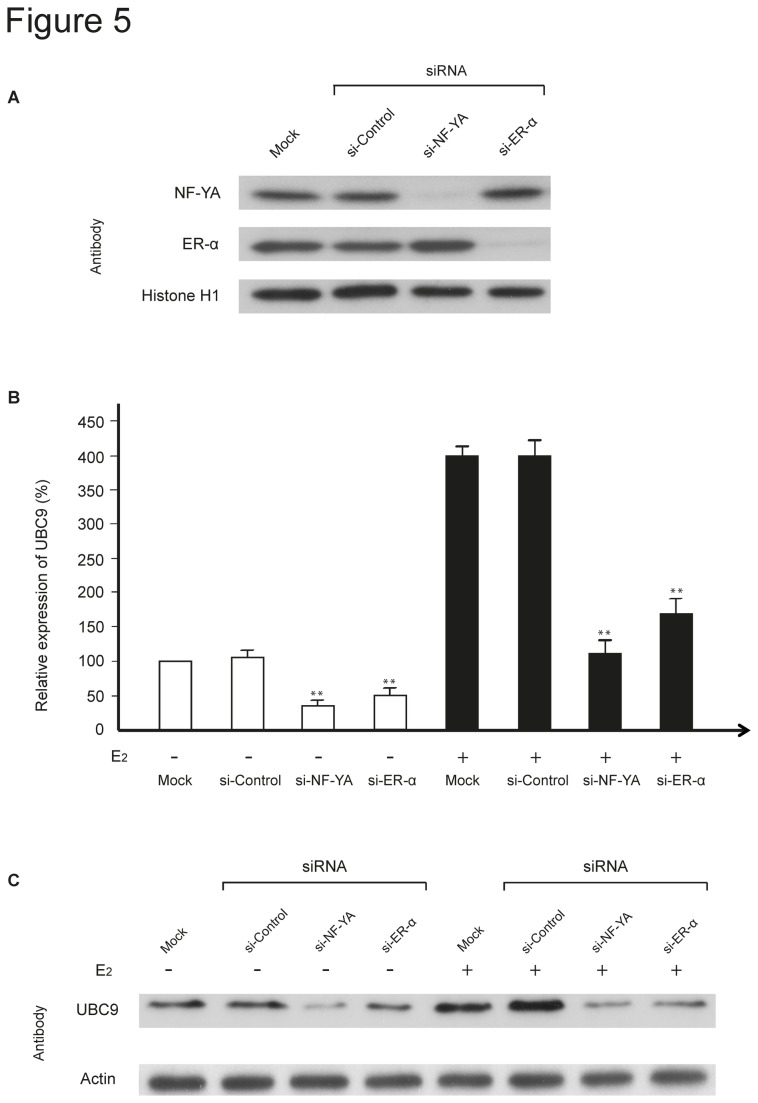
ER-α and NF-Y are essential for endogenous UBC9 expression. MCF-7 cells were transfected with 100 nM of si-ER-α or si-NF-YA or si-Control as indicated, and cultured in phenol red-free medium in the absence (white bars) or presence (black bars) of E_2_ for 48 hours. (**A**) Protein expression of ER-α and NF-YA was determined by Western blot analysis using nuclear protein extracts. Histone H1 served as a loading control. (**B**) *UBC9* mRNA expression levels were measured by quantitative RT-PCR analysis. Expression levels were normalized to GAPDH expression and relative to expression in mock transfected cells, which was arbitrarily set to 1. Data were expressed as mean ±SD obtained in four separate experiments. *P<0.05, **P<0.01 (Student’s *t*-test; si-ER-α and si-NF-YA versus si-Control). (**C**) UBC9 protein expression levels were analyzed by Western blot analysis. Actin was used as an internal protein loading control.

To provide direct evidence for the functional role of ER-α and NF-Y in *UBC9* expression, we used small interfering RNAs (siRNAs) to knock down ER-α and NF-Y *in vivo*. Transfection with si-ER-α and si-NF-YA strongly decreased ER-α and NF-Y protein levels compared to si-Control transfected or mock transfected cells (Figure 5A). Similar results were obtained after E_2_ treatment (data not shown). When ER-α or NF-Y expression was knocked down in untreated MCF-7 cells using the corresponding siRNA, UBC9 transcript levels were significantly decreased by approximately 50% and 60%, respectively, compared to si-Control treated cells (Figure 5B). The marked decreases were also obtained in E_2_ treated cells (Figure 5B). A corresponding decrease was observed on the UBC9 protein level in both untreated and E_2_ treated cells (Figure 5C). Taken together, our findings from the ChIP and siRNA knockdown experiments indicate that binding of ER-α and NF-Y to the *UBC9* promoter is essential for the transcription of the *UBC9* gene.

## Discussion

In the present study we investigated the transcriptional regulation of the human *UBC9* gene in MCF-7 and MDA-MB-231 breast cancer cells by cloning and functional characterization of its promoter. Reporter gene assays with the construct containing the 137 bp fragment of the 5’-flanking sequence of the human *UBC9* gene showed a marked basal activity in MCF-7 and MDA-MB-231 cells. We consider this fragment as the *UBC9* proximal promoter, which has one imperfect ERE for binding of ER-α and two CCAAT boxes for binding of NF-Y. In MCF-7 cells the activity of the proximal promoter fragment was enhanced 1.8-fold by E_2_ treatment, which in part explains the increase in *UBC9* mRNA levels. We further demonstrated that ER-α and NF-Y bind to these *cis*-elements in the proximal promoter and transcriptionally regulate basal and E_2_-induced *UBC9* expression *in vivo*. To our knowledge, this is the first report showing the functional role of ER-α and NF-Y in *UBC9* gene expression.

ER-α is a nuclear transcription factor that undergoes different types of post-translational modifications that regulate its transcriptional activation and/or stability [33]. There is strong evidence that estrogens play an important role in the normal physiology of the mammary gland and the development of hormone-driven breast cancer primarily through binding to its receptor (ER) [34]. Interestingly, ER-α is a target for SUMOylation, which occurs strictly in the presence of E_2_ and regulates the transcriptional activity of ER-α [16]. Moreover, SUMO is also conjugated to coregulators of ER-α, thereby modulating their ability to interact with the nuclear receptor and to activate transcription [35]. In the present study we showed that *UBC9* transcription is controlled by direct binding of ER-α to the *UBC9* proximal promoter and is affected by E_2_. As previously reported, UBC9 is an essential enzyme for SUMOylation and regulation of gene expression through different cellular pathways [1]. Furthermore, UBC9 may have multiple functional effects on ER-α and its coactivators, including SUMOylation [16] and ER associated degradation [36]. Our findings suggest crosstalk between the SUMOylation system and the ER-signalling pathway, and that their complex interaction accounts for either the correct expression or overexpression of UBC9, the latter of which is associated with the development of breast cancer. Therefore, further studies on the interaction between these two interdependent pathways, SUMOylation and estrogen signalling, are warranted to provide new insights into the mechanism underlying their involvement in breast cancer.

CCAAT boxes serve as potential binding sites for NF-Y and are frequently observed in TATA-less promoters (including the *UBC9* promoter) [37]. NF-Y consists of three subunits A, B and C, of which subunit A (NF-YA) associates with a tight dimer composed of subunits B and C, resulting in a hetero-trimeric protein that binds to DNA with high specificity and affinity in the promoter region of various genes [38–40]. In the present study we demonstrated that NF-Y is a transcription factor that activates *UBC9* transcription via binding to the two CCAAT boxes *in vivo*, and that its siRNA-mediated knockdown significantly diminished UBC9 expression on the mRNA and protein levels implying its direct functional effect on UBC9 expression. Indeed, there is evidence from previous studies that the levels of NF-Y vary in different cell types and under different growth conditions, and that its DNA-binding activities are driven by estrogens for some estrogen-induced gene expression [41,42]. These findings are in agreement with our data showing that overexpression of NF-YA stimulated *UBC9* promoter activity, especially upon treatment of MCF-7 cells with E_2_. Altogether, NF-Y may act as a key regulator for the basal expression of the *UBC9* gene in an ER-α dependent regulation pathway.

In this study the possibility of cooperative interactions between ER-α and NF-Y was conceivable for *UBC9* gene transcription through estrogen action in MCF-7 cells. Our data demonstrate that ER-α binding to the imperfect ERE motif in the *UBC9* promoter contributes to *UBC9* transactivation and that cooperative interaction with NF-Y may be required for E_2_ responsiveness. These results are also consistent with previous studies showing that the transcriptional activation of some E_2_ responsive genes may be due to stabilization of the Sp1-NF-Y-DNA complex by ER-α [41,42]. Thus, one possible function of ER-α is to stabilize the interaction of NF-Y on its binding sites. However, also other mechanisms seem to play a role in ER-α-dependent transactivation, such as activation of kinases and modulation of proteins that affect NF-Y or Sp1 action [41,42]. These non-genomic pathways activated by E_2_ have been characterized in multiple cancer cell lines including breast cancer cell lines [43,44]. The mechanisms associated with these pathways are complex and may depend on several factors including cell context and cell type [43–47]. ERs can regulate gene expression without directly binding to DNA [41,42,45], which may explain E_2_ responsiveness of the ERE mutant. We speculate that in the case of *UBC9* transcription, E_2_-dependent transactivation may involve both genomic (ER-α /NF-Y direct binding to DNA) and non-genomic (specific signal molecules) pathways of estrogen action. In other words, ER-α is the main transcription factor that specifically responds to E_2_ and NF-Y may coordinately enhance transcriptional induction by ER-α.

*UBC9* transcription probably has specific regulation patterns in different breast cancer cell lines. Interestingly, our results showed a higher UBC9 expression in ER-negative MDA-MB-231 cells than in ER-positive MCF-7 cells implying that NF-Y plays a dominant role in an “ER-free” environment. NF-YA is alternatively spliced resulting in a long and short isoform [48] and their cellular distribution could impart an important cell-specific component to gene transcriptional regulation [49]. As previously reported, both NF-YA isoforms are expressed in MDA-MB-231 cells with a higher expression of the long isoform compared to the short isoform [48,50]. In contrast, MCF-7 cells mainly express the short isoform [50]. Furthermore, there is evidence that the two NF-YA isoforms have different effects on promoter activity with the long isoform having a much stronger transactivation capability than the short form [49,51]. The amount of NF-YA recruited on the promoter also may differ between various cell types. Our ChIP data showed an approximately 2-fold higher recruitment of NF-YA on the *UBC9* promoter in MDA-MB-231 cells compared to MCF-7 cells. Taken together, the isoform and/or amount of recruited NF-YA to the promoter may explain the higher UBC9 expression in MDA-MB-231 cells. Further studies on specific expression of UBC9 in different breast cancer cells are warranted.

In this study we showed that expression of UBC9 is regulated on the transcription level through ER-α and NF-Y. Indeed, eukaryotic gene expression is regulated on many levels, including epigenetic, transcriptional, post-transcriptional, translational and post-translational. Two previous studies reported that *UBC9* expression is negatively regulated by miR-30e and miR-214 [23,52]. Thus regulation of UBC9 expression probably also occurs on the post-transcriptional level. Furthermore, another study reported that high expression of cdc2 possibly contributes to hyperphosphorylation of UBC9 in several cancers by post-translational regulation [53]. Moreover, UBC9 acetylation was considered as a key regulatory step in controlling SUMOylation of substrates [54,55]. In addition, UBC9 also directly binds to nuclear receptors like the androgen receptor [56,57], glucocorticoid receptor [58,59] and ER-α [60] and regulates their activity. It also may be possible that the nuclear receptors regulate UBC9. These findings suggest that *UBC9* expression is controlled on multiple levels *in vivo*.

Regulation of UBC9 expression in cancers is of clinical relevance. By using MCF-7 breast cancer cells overexpressing a UBC9 dominant-negative mutant (UBC9-DN), or wild type UBC9 in a mouse xenograft model, it was shown that tumors expressing the UBC9 mutant exhibited reduced growth, whereas wild type UBC9 enhanced tumor growth [21]. So far, more than 150 proteins have been identified as SUMO targets, many of which are involved in cell proliferation, differentiation and cell cycle control [61]. Therefore it is conceivable that deregulation of *UBC9* expression leading to alterations of SUMOylation-mediated cellular pathways contributes to cancer development. Moreover, there is evidence that overexpression of UBC9 affects tumor drug responsiveness. DNA isotopomerase I (topo I), which plays a role in DNA metabolism and transcription, is modified by SUMO and is targeted by anticancer drugs such as camptothecin, topotecan and irinotecan. Overexpression of UBC9-DN sensitized tumor cells to inhibitors of topo I and topo 2 as well as cisplatin, a DNA alkylating agent [26]. Additionally, a strong correlation between UBC9 levels and drug resistance in ovarian cancer and acute lymphoblastic leukemia cell lines was observed, further supporting a role of UBC9-mediated SUMOylation in tumor drug responsiveness.

In summary, our results showed that ER-α and NF-Y bind directly to the *UBC9* proximal promoter and are critical for the *in vivo* expression of this gene via transcriptional regulation. Moreover, UBC9 expression is affected by E_2_ and overexpression of ER-α and NF-Y. Our findings may contribute to a better understanding of *UBC9* regulation in MCF-7 breast cancer cells and be useful for the development of cancer therapies targeting UBC9.

## Supporting Information

Figure S1 **UBC9 expression in MCF-7 and MDA-MB-231 breast cancer cell lines.** (**A**) UBC9 mRNA expression in ER-positive MCF-7 and ER-negative MDA-MB-231 breast cancer cells. Total RNA was isolated and analysed by real-time RT-PCR. Expression levels were normalized to GAPDH expression and relative to expression in MCF-7 cells, which was arbitrarily set to 1. The data refer to results obtained in four separate experiments performed in triplicate. Bars represent the standard deviation (SD). (**B**) UBC9 protein expression in the two indicated cell lines. Total protein was extracted and analysed by Western blotting. Actin was used as an internal protein loading control.
(TIFF)

Table S1 **List of oligonucleotide sequences used in this study.**
(PDF)
