# Supplementary figures and images for: Estrogen Receptor Alpha and Nuclear Factor Y Coordinately Regulate the Transcription of the SUMO-Conjugating UBC9 Gene in MCF-7 Breast Cancer Cells
Source: PLoS One. 2013 Sep 27;8(9):e75695. doi: 10.1371/journal.pone.0075695 (PMC3785449; doi:10.1371/journal.pone.0075695)

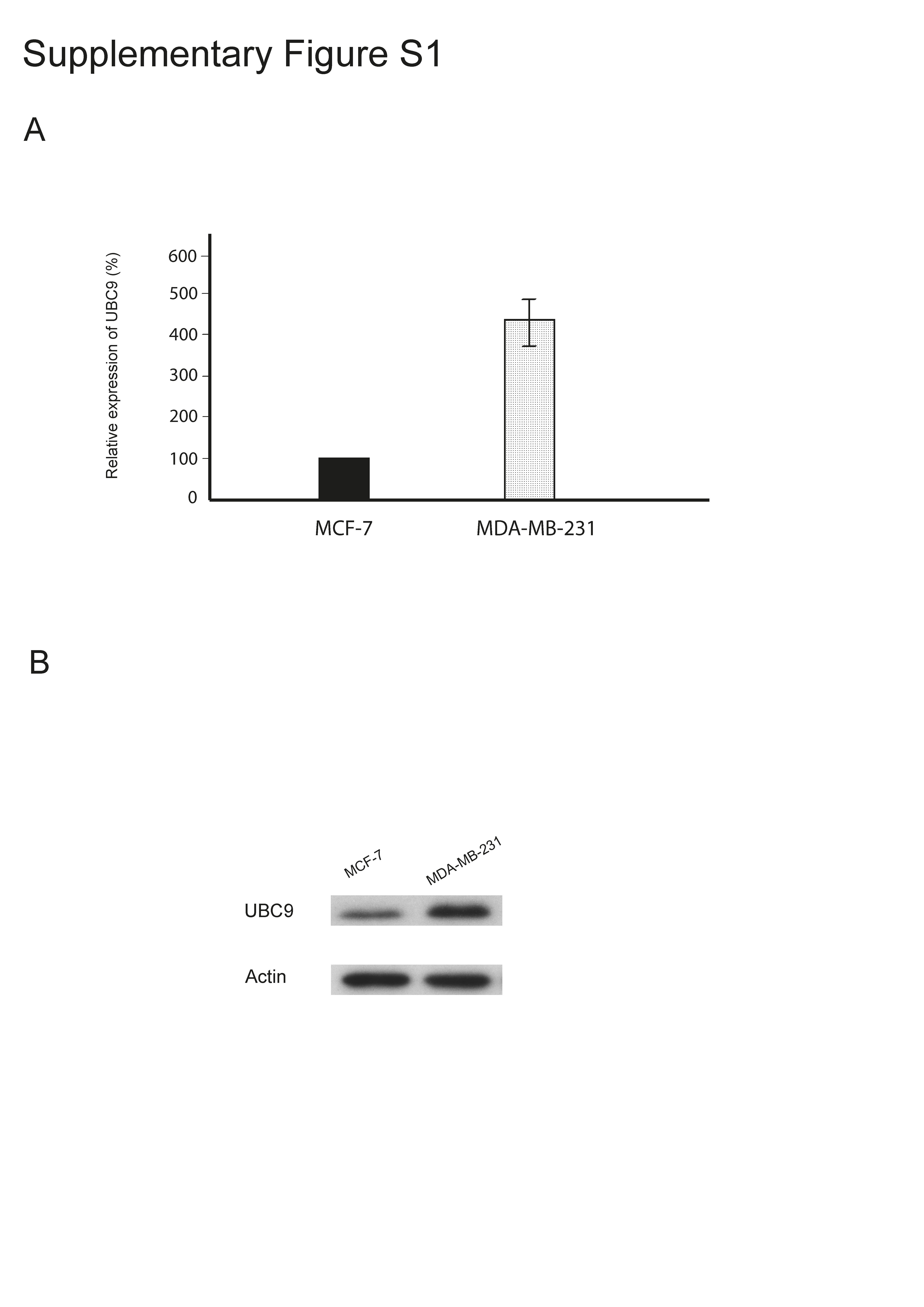

Supplement: Figure S1 — UBC9 expression in MCF-7 and MDA-MB-231 breast cancer cell lines. (A) UBC9 mRNA expression in ER-positive MCF-7 and ER-negative MDA-MB-231 breast cancer cells. Total RNA was isolated and analysed by real-time RT-PCR. Expression levels were normalized to GAPDH expression and relative to expression in MCF-7 cells, which was arbitrarily set to 1. The data refer to results obtained in four separate experiments performed in triplicate. Bars represent the standard deviation (SD). (B) UBC9 protein expression in the two indicated cell lines. Total protein was extracted and analysed by Western blotting. Actin was used as an internal protein loading control. (TIFF) [file pone.0075695.s001.tiff]
