# Supplementary material for: Estrogen Receptor Alpha and Nuclear Factor Y Coordinately Regulate the Transcription of the SUMO-Conjugating UBC9 Gene in MCF-7 Breast Cancer Cells
Source: PLoS One. 2013 Sep 27;8(9):e75695. doi: 10.1371/journal.pone.0075695 (PMC3785449; doi:10.1371/journal.pone.0075695)
Supplement: Table S1 — List of oligonucleotide sequences used in this study. (PDF) [file pone.0075695.s002.pdf]

Supplementary Table S1. List of oligonucleotide sequences used in this study

Application		Forward Primer (5'-3')	Reverse Primer (5'-3')
**Construction of reporter vectors**			
	UBC9, promoter -2392/+124	TTAGGTACCTGTGCTGGCATCTTCGCCAG	TTCAGATCTGTCACCTCTCCAGCCACAG
	UBC9, promoter -1852/+124	TTAGGTACCGCTGTGCATGGCTTCCAGG	TTCAGATCTGTCACCTCTCCAGCCACAG
	UBC9, promoter -1310/+124	TTAGGTACCGGAGAGAATCAGTGAGG	TTCAGATCTGTCACCTCTCCAGCCACAG
	UBC9, promoter -404/+124	TTAGGTACCACACCGTGACCTTGGCTG	TTCAGATCTGTCACCTCTCCAGCCACAG
	UBC9, promoter -137/+124	TTAGGTACCGAGCATCCACACTTCTCCTCC	TTCAGATCTGTCACCTCTCCAGCCACAG
	UBC9, promoter -5/+124	TTAGGTACCCGCTGAACTCGCGGGAGCG	TTCAGATCTGTCACCTCTCCAGCCACAG
**Site-directed mutagenesis**			
	pGL -137/+124, ERE-Mut	CCACACTTCTCCTCCCGTC*GGTTT*TGCTCGCATTCG	CGAATGCGAGCA*AA*ACCGACGGGAGGAGAAGTGTGG
	pGL -137/+124, iCCAAT box-Mut	TTTTGTACGGGGTCAGCTCATT*TT*GCGGATGAGAAGGTG	CACCTTCTCATCCGC*AA*AATGAGCTGACCCCGTACAAAA
	pGL -137/+124, CCAAT box-Mut	CGCCCCGAACTGGCC*TT*TTGCAAGGGGTTTCA	TGAAACCCCTTGCAA*AA*GGCCAGTTCGGGGCG
**qRT-PCR of *UBC9* mRNA**			
	UBC9	TCGAACCACCATTATTTCAC	TGTTTGATTGTGATGGCTG
	GAPDH	CATGTTCCAATATGATTCCAC	CCTGGAAGATGGTGATG
**Real-time PCR of ChIP**			
	UBC9, promoter -137/+124	AGCATCCACACTTCTCCTC	TTGCAATTGGCCAGTTC
	UBC9, exon 7, negative control region	TCGAACCACCATTATTTCAC	TGTTTGATTGTGATGGCTG

Note: Recognition sequences for the restriction enzyme (*Kpn*I or *Bgl*II) are underlined. Mutated bases are both underlined and italic.
Abbreviation list: *UBC9* gene (human ubiquitin-conjugating enzyme *UBE2I* gene, NCBI Gene ID: 7329); GAPDH (Human glyceraldehyde-3-phosphate dehydrogenase, NCBI Gene ID: 2597)
